# N^6^-methyladenosine-modified CircRNA-SORE sustains sorafenib resistance in hepatocellular carcinoma by regulating β-catenin signaling

**DOI:** 10.1186/s12943-020-01281-8

**Published:** 2020-11-23

**Authors:** Junjie Xu, Zhe Wan, Minyue Tang, Zhongjie Lin, Shi Jiang, Lin Ji, Kirill Gorshkov, Qijiang Mao, Shunjie Xia, Dong Cen, Junhao Zheng, Xiao Liang, Xiujun Cai

**Affiliations:** 1 Department of General Surgery, Sir Run-Run Shaw Hospital, Zhejiang University School of Medicine, Hangzhou, 310016 China; 2 Key Laboratory of Laparoscopic Technology of Zhejiang Province, Hangzhou, China; 3 Zhejiang Minimal Invasive Diagnosis and Treatment Technology Research Center of Severe Hepatobiliary Disease, Hangzhou, China; 4 Zhejiang Research and Development Engineering Laboratory of Minimally Invasive Technology and Equipment, Hangzhou, China; 5 Zhejiang University Cancer Center, Hangzhou, China; 6 Department of Reproductive Endocrinology, Women’s Hospital, Zhejiang University School of Medicine, Hangzhou, 310006 China; 7 National Center for Advancing Translational Sciences, National Institutes of Health, 9800 Medical Center Drive, Bethesda, MD 20892 USA

**Keywords:** Sorafenib resistance, Hepatocellular carcinoma, Circular RNA, m^6^A

## Abstract

**Background and aims:**

Accumulating evidence suggests that the primary and acquired resistance of hepatocellular carcinoma (HCC) to sorafenib is mediated by multiple molecular, cellular, and microenvironmental mechanisms. Understanding these mechanisms will enhance the likelihood of effective sorafenib therapy.

**Methods:**

In vitro and in vivo experiments were performed and clinical samples and online databases were acquired for clinical investigation.

**Results:**

In this study, we found that a circular RNA, circRNA-SORE, which is up-regulated in sorafenib-resistant HCC cells, was necessary for the maintenance of sorafenib resistance, and that silencing circRNA-SORE substantially increased the efficacy of sorafenib-induced apoptosis. Mechanistic studies determined that circRNA-SORE sequestered miR-103a-2-5p and miR-660-3p by acting as a microRNA sponge, thereby competitively activating the Wnt/β-catenin pathway and inducing sorafenib resistance. The increased level of circRNA-SORE in sorafenib-resistant cells resulted from increased RNA stability. This was caused by an increased level of N^6^-methyladenosine (m^6^A) at a specific adenosine in circRNA-SORE. In vivo delivery of circRNA-SORE interfering RNA by local short hairpin RNA lentivirus injection substantially enhanced sorafenib efficacy in animal models.

**Conclusions:**

This work indicates a novel mechanism for maintaining sorafenib resistance and is a proof-of-concept study for targeting circRNA-SORE in sorafenib-treated HCC patients as a novel pharmaceutical intervention for advanced HCC.

**Supplementary Information:**

The online version contains supplementary material available at 10.1186/s12943-020-01281-8.

## Introduction

Hepatocellular carcinoma (HCC) is the most common cancer and the leading cause of cancer-associated mortality worldwide. Surgical resection is the first choice treatment for HCC, but not for advanced HCC patients [1]. For unresectable HCC, treatment options include transplantation, ablation, transarterial chemoembolization, targeted therapies and immunotherapies. Sorafenib, a multikinase inhibitor, is a first-line targeted drug approved by the US Food and Drug Administration for advanced HCC [2]. According to the results of the Sorafenib Hepatocellular Carcinoma Assessment Randomized Protocol (SHARP) trial, sorafenib can prolong survival of HCC patients. However, drug resistance limits its efficacy. Although some mechanisms have been reported for sorafenib resistance, such as epithelial-mesenchymal transition, the proliferation of cancer stem cells, and metabolic reprogramming, the exact cause is still elusive [3]. Therefore, understanding the underlying molecular basis of HCC sorafenib resistance and developing mechanism-based therapies are urgently needed.

Circular RNAs (circRNAs) have a circular configuration through a typical 5′ to 3′-phosphodiester bond and are recognized as a class of functional non-coding RNAs (ncRNAs). CircRNAs regulate biological processes by mediating alternative splicing (AS) of RNAs, cis-regulation of transcription, and by acting as competing endogenous RNAs (ceRNAs) [4]. Importantly, many circRNAs are involved in cell proliferation, differentiation, apoptosis and invasion during tumor progression [5, 6]. Recent studies show that the circRNA, CDR1as, can accelerate the proliferation and migration of HCC cells by promoting the expression of AFP via sponging miR-1270 [7], while circRHOT1 can inhibit HCC development and progression via recruiting TIP60 to initiate NR2F6 expression [8]. Circ_0003418 increases the sensitivity of HCC cells to cisplatin by inhibiting the Wnt/β-catenin pathway [9]. However, the roles of circRNAs in HCC sorafenib resistance remain unknown.

N6-methyladenosine (m^6^A) is the most prevalent internal modification associated with eukaryotic mRNAs and ncRNAs and it influences many steps of mRNA metabolism, including splicing, export, translation, and stability [10]. The m^6^A modification has been implicated in various cellular and physiological events, including carcinogenesis [11]. In acute lymphoblastic leukemia (ALL), levels of METTL3 (a component of the methyltransferase complex that catalyzes adenosine methylation) increase in AML patients and it plays an oncogenic role by inhibiting cell differentiation and apoptosis, and promoting cell proliferation through increased c-MYC, BCL2, and PTEN translation [12]. In breast cancer, mammalian hepatitis B X-interacting protein (HBXIP) enhances the expression of METTL3 by suppressing the tumor suppressor, let-7 g, and forms a positive feedback loop to enhance the level of HBXIP by facilitating m^6^A modification of mRNA, which promotes cell proliferation [13]. In HCC, m^6^A modification also regulates the progress of oncogenesis. SIRT1 promotes HCC by increasing the overall m^6^A modification level to attenuate the expression of the tumor suppressor, GNAO1 [14]. However, the functions of m^6^A modification in HCC sorafenib resistance remain elusive. Moreover, the roles of m^6^A-modified circRNAs in HCC sorafenib resistance also need further investigation.

In the present study, we found that circRNA-SORE (also named circRNA_104,797 and circ_0087293) was up-regulated in sorafenib-resistant HCC cells, and was necessary for the maintenance of sorafenib resistance. By acting as a ceRNA and sequestering miR-103a-2-5p and miR-660-3p, circRNA-SORE competitively activates the Wnt/β-catenin pathway and promotes sorafenib resistance. The increased levels of circRNA-SORE in sorafenib-resistant HCC are due to its increased stability resulting from increased N^6^-methyladenosine (m^6^A) levels of a specific adenosine in circRNA-SORE. These results provide a novel mechanism for maintaining sorafenib resistance and demonstrate a proof-of-concept for targeting circRNA-SORE in sorafenib-treated HCC patients as a novel pharmaceutical intervention for advanced HCC.

## Materials and methods

### RNA pull-down

For biotinylated microRNA (miRNA) pull-down assays, biotin-miRNA-transfected HepG2-SR cell lysates were incubated with MyOne™ Dynabead® Streptavidin C1 (Invitrogen, Grand Island, NY, USA) at 4 °C for 1 h. For biotinylated probe pull-down assays, washed MyOne™ Dynabead® Streptavidin C1 (Invitrogen, USA) was incubated with cell lysate at 4 °C for 1 h for preclearance. The 3′ biotin-labeled circRNA-SORE probe (5′-GAGTTGTTGCTGCTTGATGGAGTC-3′-biotin) was synthesized by Tsingke Biotech and incubated with the beads at room temperature for 10 min for immobilization. Then, the biotinylated beads were incubated with HepG2-SR cell lysate at 4 °C overnight. The biotinylated beads were magnetically separated and washed five times. For qRT-PCR assays, Trizol reagent was used to extract total RNA from the beads.

### Silencing and overexpression of circRNA-SORE

circRNA-SORE siRNA targeting the junction region of the circRNA-SORE sequence was synthesized by RiboBio (Guangzhou, China). Transfection of plasmids, siRNA, or miRNA mimics or inhibitors (RiboBio, Guangzhou, China) was performed using Lipofectamine® 3000 (Invitrogen, USA) according to the manufacturer’s protocol. The sequences of siRNAs against specific targets are listed in Table S1.

The sh-circRNA-SORE sequence (same as the sicircRNA-SORE sequence) was cloned into GV248. The backbone of pLCDH-ciR (Geneseed, Guangzhou, China) was used to construct the circRNA-SORE over-expression plasmid (OE-circRNA-SORE). The linear sequence of circRNA-SORE together with the flanking circRNA-inducing sequence was amplified and subcloned into EcoRI and BamHI sites of pLCDH-ciR, and termed pLCDH-ciR-SORE. The primers used were as follows:

Primer-F: 5′-CGGAATTCTGAAATATGCTATCTTACAGCAGCAACAACTCCAGGCCCA-3′.

Primer-R: 5′-CGGGATCCTCAAGAAAAAATATATTCACCTTGATGGAGTCTCTGTCTC-3′.

pLCDH-circRNA-SORE-M has a mutated circRNA-SORE m^6^A site. The sequences of mutant constructs are listed in Table S2. GV248-shcircRNA-104797, pLCDH-ciR, pLCDH-ciR-SORE, the psAX2 packaging plasmid, and the pMD2G envelope plasmid, were transfected into 293 T cells using the standard calcium phosphate transfection method to obtain lentiviral supernatant. Forty-eight hours after transfection, viruses were collected and concentrated by density gradient centrifugation, then frozen at − 80 °C for later use.

### Tissue samples

A total of 92 randomly selected HCC clinical samples were collected from the Department of General Surgery, Sir Run-Run Shaw Hospital, Zhejiang University, starting in February 2006, with all patients providing informed consent for the use of their tissues for scientific research. Another 40 tissue samples were obtained from randomly selected sorafenib-treated HCC patients to detect circRNA-SORE expression and for β-catenin immunohistochemistry staining. We reviewed pathology records to identify samples with confirmed HCC. Low and high circRNA-SORE expression levels were cut off by median expression values. The current study conformed to the principles of the Declaration of Helsinki and was approved by the Institutional Review Board of the Sir Run-Run Shaw Hospital.

Additional methods information can be found in Supplementary Materials.

## Results

### Elevated levels of circRNA-SORE in sorafenib-resistant HCC

**Fig. 1:**
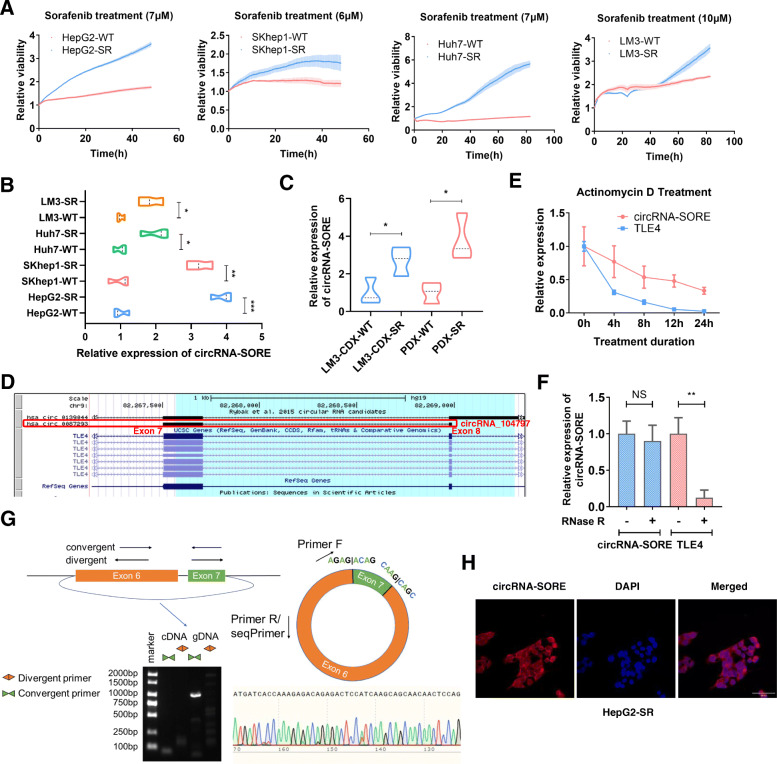
Elevated levels of circRNA-SORE in sorafenib-resistant HCC. **a** Four sorafenib-resistant HCC cell lines (HepG2-SR, SKhep1-SR, Huh7-SR and LM3-SR) were established and confirmed using the Real-time Cell Analysis xCELLigence System. Curves demonstrate the growth rate of naïve and sorafenib-resistant cell lines. **b** qPCR analysis of circRNA-SORE in the four sorafenib-resistant cell lines compared with their parental control cells. **c** qPCR analysis of circRNA-SORE in LM3-CDX-SR and PDX-SR tumor tissues compared with control tumor tissues. CDX: cell derived xenograft, PDX: patient derived xenograft. **d** circRNA-SORE information from the UCSC Genome Browser. **e** Time-course of relative circRNA-SORE and *TLE4* expression in HepG2-SR cells treated with actinomycin d (10 μg/mL). **f** qPCR analysis of circRNA-SORE and *TLE4* in HepG2, LM3 and SKhep1 cells with and without RNase R treatment for 30 min at 37 °C. **g** Verification of the circular nature of circRNA-SORE using divergent and convergent primers. Top, schematic illustration of the circRNA-SORE locus with specific primers. Bottom left, RT-PCR products using divergent primers showing circularization of circRNA-SORE. gDNA, genomic DNA. Bottom right, Sanger sequencing of circRNA-SORE. **h** Localization of circRNA-SORE (red) in HepG2-SR cells using fluorescence in situ hybridization. Cell nuclei were counterstained with DAPI (blue). Scale bar, 20 μm. Three different independent experiments with three technical repetitions were performed. Data are expressed as the mean ± SEM. Statistical analyses used Student’s t-test, and *p* < 0.05 was considered statistically significant. * *p* < 0.05, ** *p* < 0.01, and *** *p* < 0.001. NS; not statistically significant

To characterize HCC sorafenib resistance in vitro, we established four HCC sorafenib-resistant (SR) cell lines (HepG2-SR, SKhep1-SR, Huh7-SR and LM3-SR) and evaluated their resistance using the Real-time Cell Analysis xCELLigence System. As shown in Fig. 1a, sorafenib-resistant cell lines were less susceptible to sorafenib-killing than wild-type cell lines and, therefore, exhibited a faster growth rate with sorafenib treatment. We previously found that circRNA-SORE (a circular RNA up-regulated in sorafenib-resistant HCC cells) was upregulated in sorafenib-resistant HCC (unpublished observation) and circRNA-SORE was consistently upregulated in the sorafenib-resistant cell lines (Fig. 1b). Moreover, we confirmed its upregulation in sorafenib-resistant cell-derived xenograft (CDX) and PDX models (Fig. 1c), indicating the importance of circRNA-SORE in the induction and maintenance of HCC sorafenib resistance.

According to the UCSC Genome Browser, circRNA-SORE is generated from back-splicing of the 7th and 8th exons of the *TLE4* gene (Fig. 1d). We treated cells with actinomycin D (an RNA synthesis inhibitor) to confirm that circRNA-SORE was more stable than its host *TLE4* mRNA (Fig. 1e). circRNA-SORE was resistant to RNase R (a linear RNA degrader), but linear *TLE4* mRNA was not (Fig. 1f). We further validated the nature of circRNA-SORE by RT-PCR using convergent and divergent primers, which yielded a specific divergent band from cDNA, but not gDNA (Fig. 1g). Sanger sequencing of the PCR product showed the circRNA-SORE sequence and the sequence in circBase to be the same (Fig. 1g). The functions of a circRNA can be influenced by its cellular localization. Fluorescence in situ hybridization (FISH) for circRNA-SORE demonstrated its cytoplasmic localization (Fig. 1h).

Overall, we identified a circular RNA related to HCC sorafenib resistance, circRNA-SORE, that was localized in the cytoplasm and up-regulated in sorafenib-resistant cells.

### circRNA-SORE is vital for sustaining sorafenib resistance

**Fig. 2:**
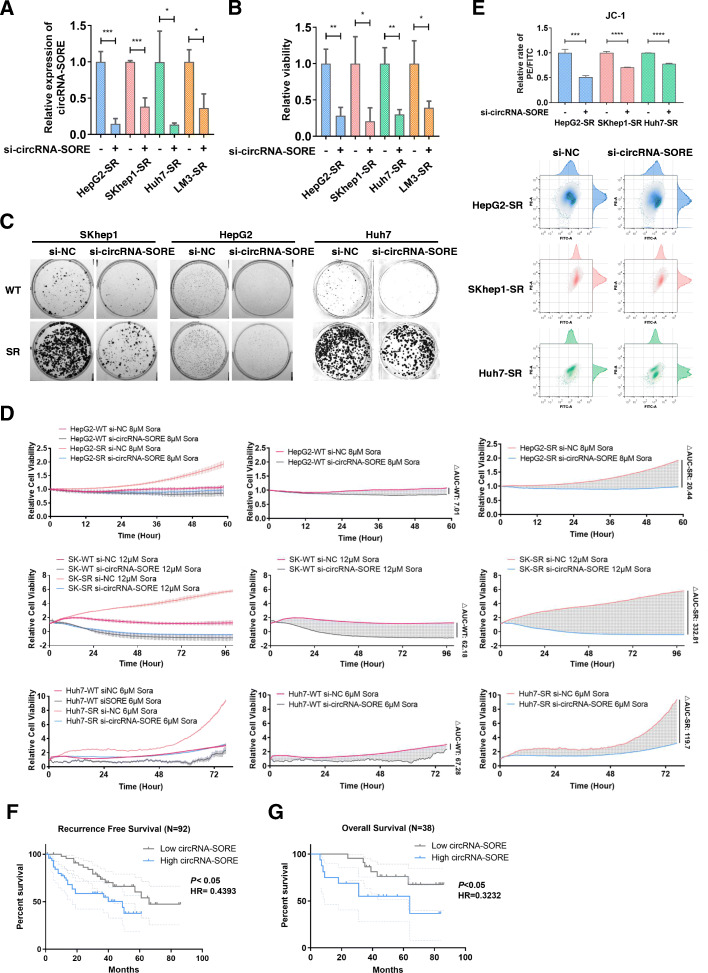
circRNA-SORE is vital for sustaining sorafenib resistance. **a** qPCR analysis of circRNA-SORE in four HCC sorafenib-resistant cell lines with and without si-circRNA-SORE under sorafenib treatment for 72 h. **b** Relative cell viability of four HCC sorafenib-resistant cell lines with and without si-circRNA-SORE under sorafenib treatment for 72 h. **c** Images of colony formation assay wells for three HCC sorafenib-resistant cell lines and their parental cell lines with and without si-circRNA-SORE under sorafenib treatment for 72 h. **d** Wild-type HCC cells and sorafenib-resistant cells transfected with si-circRNA-SORE or negative control siRNA (si-NC) were treated with sorafenib. Cell viability was recorded by the Real-time Cell Analysis xCELLigence System. ΔAUC (difference in the area under curve) = (AUC NC) – (AUC si-SORE). **e** Analysis of apoptosis in HepG2-SR, SKhep1-SR and Huh7-SR cells with or without si-circRNA-SORE by JC-1 assays. **f** Kaplan-Meier survival analysis for HCC patients with low and high circRNA-SORE expression. Low and high circRNA-SORE expression was cut off by the median expression. **g** Kaplan-Meier survival analysis for HCC patients treated with Sorafenib with low and high circRNA-SORE expression. The low and high circRNA-SORE expression was cut off by the median expression. *p* < 0.05 was considered statistically significant. Three different independent experiments with three technical repetitions were performed. Data are expressed as the mean ± SEM. Statistical analyses used Student’s t-test and Kaplan-Meier survival analysis. *p* < 0.05 was considered statistically significant. * *p* < 0.05, ** *p* < 0.01, *** *p* < 0.001 and **** *p* < 0.0001

To clarify the function of circRNA-SORE in sorafenib resistance, we knocked down circRNA-SORE using siRNA targeting the junction region of the circRNA sequence. Importantly, sorafenib resistance was significantly compromised by circRNA-SORE knock-down in the HCC sorafenib-resistant cell lines (Fig. 2a, b). Colony formation assays showed similar results (Fig. 2c), indicating a critical role for circRNA-SORE in HCC sorafenib resistance. Real-time cell analyses showed significantly reduced viability of circRNA-SORE knock-down sorafenib-resistant cells, but not of wild-type cells (Fig. 2d), indicating that circRNA-SORE likely functions in a specific sorafenib-resistant context. JC-1 assays further revealed increased apoptosis in sorafenib-resistant cells upon circRNA-SORE knock-down (Fig. 2e). Importantly, HCC patients with lower circRNA-SORE expression levels had better recurrence free survival and overall survival than patients with higher circRNA-SORE expression levels (Fig. 2f, g). Thus circRNA-SORE is potentially important for HCC prognosis.

Our results show that circRNA-SORE is essential for sustaining sorafenib resistance and that silencing circRNA-SORE substantially increased the efficacy of sorafenib by inducing tumor cell apoptosis.

### circRNA-SORE sustains sorafenib resistance by acting as an miRNA sponge for miR-103a-2-5p and miR-660-3p

**Fig. 3:**
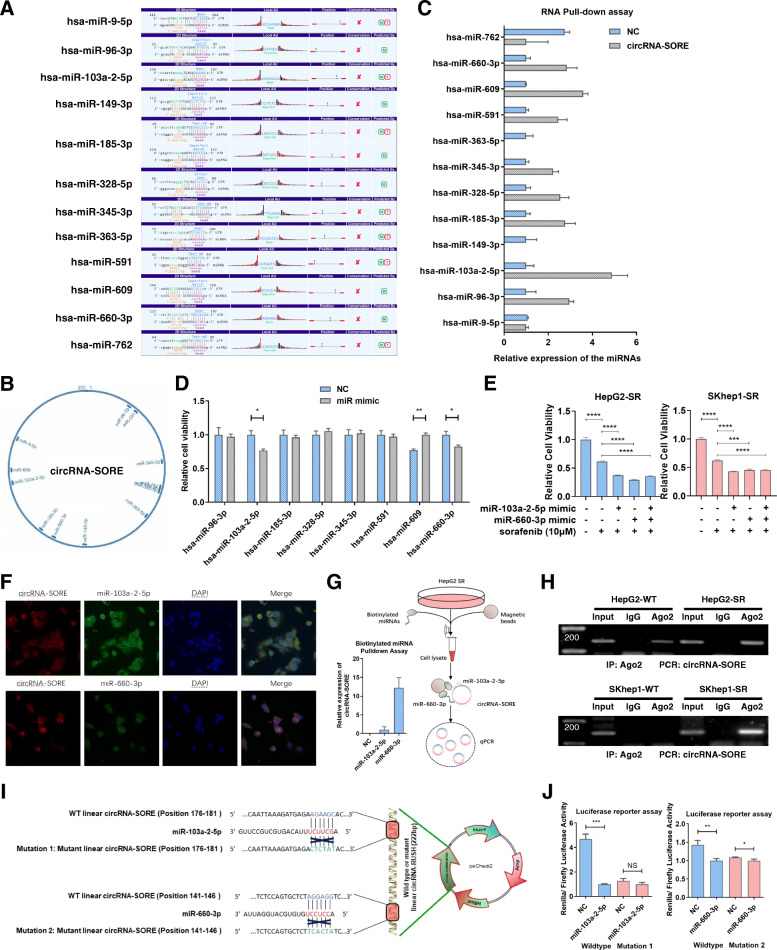
circRNA-SORE sustains sorafenib resistance by acting as an miRNA sponge for miR-103a-2-5p and miR-660-3p. **a** Predicted miRNA interactions with circRNA-SORE using Arraystar’s miRNA target prediction software based on TargetScan & miRanda. **b** Map of circRNA-SORE and its predicted miRNAs interactions. **c** qPCR analysis for predicted miRNAs pulled-down by circRNA-SORE. **d** CCK-8 assays for HepG2-SR cells transfected with miRNA mimics and controls. **e** CCK-8 assays for HepG2-SR and SKhep1-SR cells transfected with miR-103a-2-5p, miR-660-3p mimics and controls with or without sorafenib treatment. **f** The co-localization of circRNA-SORE (red) and miR-103a-2-5p/miR-660-3p (green) was observed by fluorescence in situ hybridization (FISH) in HepG2-SR cells. Cell nuclei were counterstained with DAPI (blue). Scale bar: 5 μm. **g** Left: qPCR analysis of circRNA-SORE in the pull-down lysates of biotinylated miR-103a-2-5p or miR-660-3p. Right: workflow for the biotinylated miRNA pull-down assays. **h** Ago2 radioimmunoprecipitation assays for circRNA-SORE in wild-type HCC cells and sorafenib-resistant cells. **i–j** Dual *Renilla* and firefly luciferase reporter assays using the linear form of wild-type and mutant circRNA-SORE in HepG2-SR cells transfected with NC or miR-103a-2-5p/miR-660-3p miRNA mimics. Three different independent experiments with three technical repetitions were performed. Data are expressed as the mean ± SEM. Statistical analyses used Student’s t-test, and *p* < 0.05 was considered statistically significant. * *p* < 0.05, ** *p* < 0.01, *** *p* < 0.001 and **** *p* < 0.0001. NS; not statistically significant

circRNAs can function in numerous ways, including by sponging miRNAs, interacting with proteins, promoting gene transcription, and encoding peptides [15]. Given that circRNA-SORE localizes to the cytoplasm, as described above, we hypothesized that miRNA sponge activity could be a possible mechanism for its functional effects. circRNA-miRNA interactions were predicted using Arraystar’s miRNA target prediction software based on TargetScan [16] & miRanda [17] (Fig. 3a, b). Among these predicted miRNAs, miR-96-3p, miR-103a-2-5p, miR-185-3p, miR-328-5p, miR-345-3p, miR-591, miR-609, and miR-660-3p were found to be associated with circRNA-SORE (Fig. 3c). By transfecting mimics of these miRNAs, we found that miR-103a-2-5p and miR-660-3p reduced sorafenib resistance in HepG2-SR cells (Fig. 3d, e). RNA-FISH for circRNA-SORE further revealed the colocalization of circRNA-SORE with miR-103a-2-5p and miR-660-3p (Fig. 3f). Consistently, biotinylated miRNA pull-down assays showed significant enrichment of circRNA-SORE with miR-103a-2-5p and miR-660-3p (Fig. 3g). Ago2 immunoprecipitation assays detected circRNA-SORE in the RNA-induced silencing complex (RISC) in sorafenib-resistant cells and not in wild-type cells (Fig. 3h). Moreover, according to the predicted miRNA binding sites on circRNA-SORE, luciferase reporter assays of wild-type and mutant linear forms of circRNA-SORE showed that miR-103a-2-5p and miR-660-3p could specifically target the wild-type linear form of circRNA-SORE resulting in decreased *Renilla* luciferase activity, but not the mutant 1 or 2 forms (Fig. 3i, j). These results suggested a miRNA sponging role of circRNA-SORE for miR-103a-2-5p and miR-660-3p in sorafenib-resistant HCC.

**Fig. 4:**
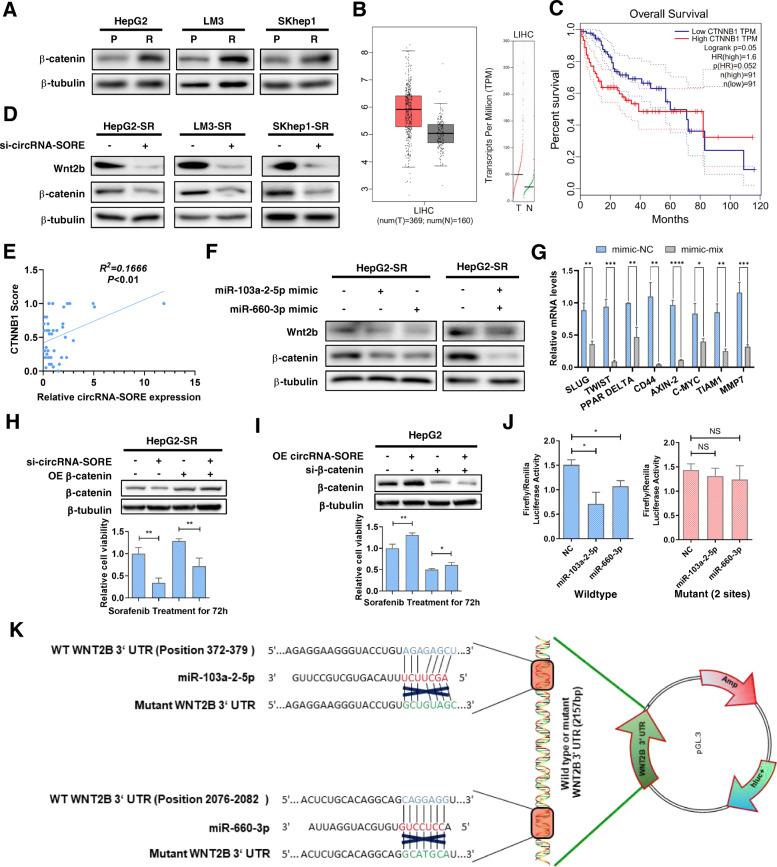
miR-103a-2-5p and miR-660-3p regulate Wnt2b/β-catenin signaling*.* **a** Western blotting for β-catenin and β-tubulin in three sorafenib-resistant cell lines compared with their parental control cell lines. **b** Box-plot (left) and gene expression level (right) of CTNNB1 expression in TCGA HCC tumor and matched TCGA normal liver tissues along with GTEx data. **c** Overall survival analysis of HCC patients (*N* = 182) from TCGA project with high or low CTNNB1 expression levels (defined by RNA sequencing with group cutoff in 75%/25% quartile). **d** Western blot for Wnt2b, β-catenin and β-tubulin in three sorafenib-resistant cell lines with or without si-circRNA-SORE. **e** Correlation of relative RNA expression of circRNA-SORE and protein expression of CTNNB1 by immunohistochemistry. The slides were reviewed and scored by an experienced pathologist without the knowledge of patient outcome. **f** Western blot for Wnt2b, β-catenin, β-tubulin with or without miR-103a-2-5p/miR-660-3p mimics in HepG2-SR. **g** qPCR analysis for downstream genes of β-catenin. **h** Western blot for β-catenin and β-tubulin (upper) and CCK8 assays (lower) for HepG2-SR cells transfected with si-circRNA-SORE and over-expressing β-catenin. **i** Western blot for β-catenin and β-tubulin (upper) and CCK8 assays (lower) for HepG2 cells transfected with si-β-catenin and over-expressing circRNA-SORE. **j-k** *Renilla* and firefly dual luciferase reporter assay using Wnt2b 3′-UTR constructs containing wild-type or mutant seed sequences in HepG2-SR cells transfected with negative control (NC) or miR-103a-2-5p/miR-660-3p miRNA mimics. Three different independent experiments with three technical repetitions were performed. Data are expressed as the mean ± SEM. Statistical analyses used Student’s t-test, and *p* < 0.05 was considered statistically significant. * *p* < 0.05 and ** *p* < 0.01. NS; not statistically significant

To elucidate the downstream mechanisms of circRNA-SORE, several recently reported sorafenib-resistance-related genes were screened in parental and sorafenib-resistant HCC cell lines. Among these, β-catenin was consistently up-regulated in sorafenib-resistant cell lines (Fig. 4a). Analysis of HCC patient RNA-seq expression data from The Cancer Genome Atlas (TCGA) project [18] indicated increased *CTNNB1* expression in tumor compared to normal liver tissue (Fig. 4b). Importantly, survival analysis showed that patients with higher *CTNNB1* expression had worse overall survival compared with those with lower *CTNNB1* expression (Fig. 4c with group cutoffs at 75%/25%). Furthermore, silencing circRNA-SORE in three HCC sorafenib-resistant cell lines showed that Wnt2b/β-catenin signals may be closely linked with circRNA-SORE function because Wnt2b and β-catenin were significantly down-regulated upon circRNA-SORE silencing (Fig. 4d). Moreover, we measured circRNA-SORE expression in our collection of HCC tumor samples and found that the immunohistochemistry score of β-catenin was significantly correlated with the expression of circRNA-SORE (Fig. 4e), indicating that circRNA-SORE could regulate Wnt2b/β-catenin signals. Consistently, miR-103a-2-5p and miR-660-3p alone and in combination significantly decreased the expression of Wnt2b and β-catenin (Fig. 4f), and miR-103a-2-5p and miR-660-3p in combination suppressed the transcription of β-catenin downstream signals (Fig. 4g). To further assess the role of β-catenin in mediating circRNA-SORE functions, we performed rescue assays by over-expressing and silencing β-catenin. These assays showed that β-catenin could partially rescue the effect of circRNA-SORE (Fig. 4h, i), indicating that circRNA-SORE sustains sorafenib resistance by regulating Wnt2b/β-catenin signals.

We further hypothesized that miR-103a-2-5p and miR-660-3p inhibit the expression of Wnt2b by targeting its 3′-untranslated region (3′-UTR). Reporter assays demonstrated that both miR-103a-2-5p and miR-660-3p decreased the luciferase activity of the wild-type reporter, while no significant changes were found using mutant reporters (Fig. 4j, k).

Our results show that circRNA-SORE can specifically sponge miR-103a-2-5p and miR-660-3p to act as a ceRNA, thereby competitively activating the Wnt/β-catenin pathway to promote sorafenib resistance.

### circRNA-SORE is modulated by m^6^A RNA methylation

**Fig. 5:**
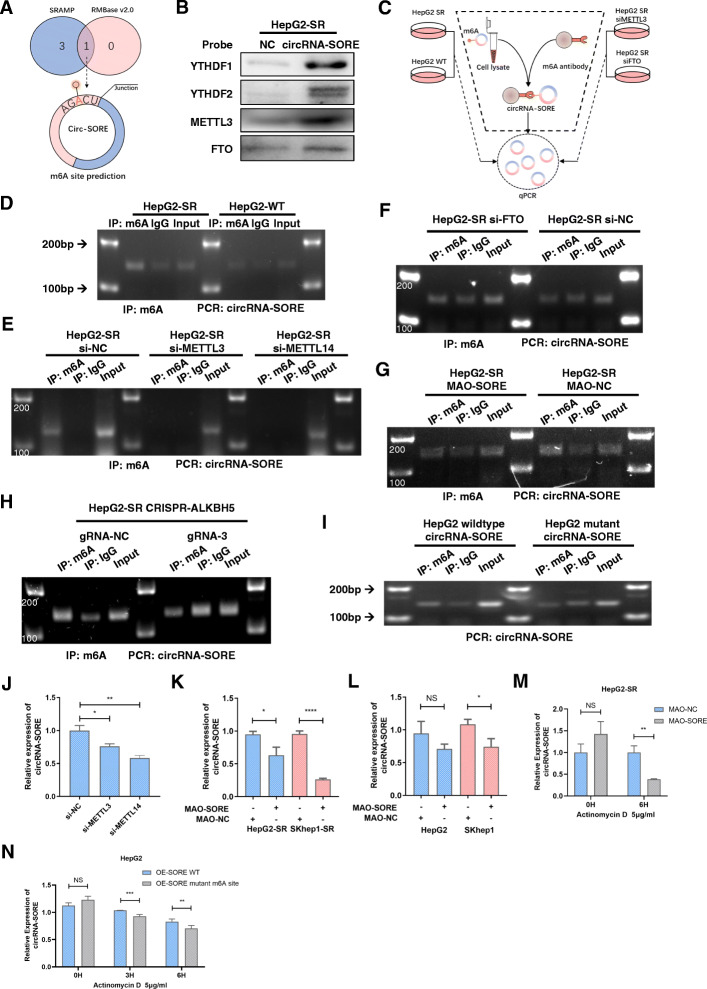
circRNA-SORE is modulated by m^6^A RNA methylation. **a** Predicted m^6^A site in circRNA-SORE from overlapping results of a sequence-based N6-methyladenosine (m^6^A) modification site predictor (SRAMP) and RMBase v2.0. **b** RNA pulldown assays and western blotting for YTHDF1/2, METTL3, and FTO using a circRNA-SORE probe. **c** Flow diagram of m6A-specific immunoprecipitation (MeRIP) assays. **d** MeRIP assays for m^6^A-modified circRNA-SORE in HepG2-SR and HepG2-WT cell lines. **e** MeRIP assays for m^6^A-modified circRNA-SORE in HepG2-SR cells transfected with si-NC, si-METTL3 or si-METTL14. **f** MeRIP assays for m^6^A-modified circRNA-SORE in HepG2-SR cells transfected with si-FTO or si-NC. **g** MeRIP assays for m^6^A-modified circRNA-SORE in HepG2-SR cells transfected with MAO-SORE or MAO-NC. MAO-SORE: sequence-specific morpholino antisense oligos blocking the m^6^A site in circRNA-SORE. **h** MeRIP assays for m^6^A-modified circRNA-SORE in HepG2-SR cells programmable edited by CRISPR-Cas9-ALKBH5 conjugates. gRNA-3: targeting the predicted m^6^A site in circRNA-SORE; gRNA-NC: negative control gRNA. **i** MeRIP assays for m^6^A-modified circRNA-SORE in HepG2-SR cells over-expressing circRNA-SORE with or without the m^6^A site mutated. **j** qPCR analysis of circRNA-SORE in HepG2-SR cells transfected with si-METTL3, si-METTL14 or si-NC. **k** qPCR analysis of circRNA-SORE in HepG2-SR and SKhep1-SR cells transfected with MAO-SORE or MAO-NC. **l** qPCR analysis of circRNA-SORE in HepG2 and SKhep1 cells transfected with MAO-SORE or MAO-NC. **m** qPCR analysis of circRNA-SORE in HepG2-SR cells transfected with MAO-SORE or MAO-NC under actinomycin D (5 μg/mL) treatment for 0 and 6 h. **n** qPCR analysis of circRNA-SORE in HepG2 cells over-expressing circRNA-SORE with or without the m^6^A site mutated under actinomycin D (5 μg/mL) treatment for 0, 3 and 6 h. Three different independent experiments with three technical repetitions were performed. Data are expressed as the mean ± SEM. Statistical analyses used Student’s t-test, Kaplan-Meier survival analysis and the log-rank test. *p* < 0.05 was considered statistically significant. * p < 0.05, ** p < 0.01, *** *p* < 0.001 and **** *p* < 0.0001. NS; not statistically significant

circRNA-SORE is likely generated through pre-mRNA back-splicing of the 7th and 8th exons of the *TLE4* transcript. However, the detailed mechanisms controlling circRNA-SORE levels remain elusive. m^6^A is the most abundant base modification of RNA and it might play a role in circRNA biogenesis [19, 20]. In particular, m^6^A-containing circRNAs are reported to be subjected to endoribonucleolytic cleavage by YTHDF2-HRSP12-RNase P/MRP [10]. The modification preferentially occurs in the consensus motif ‘RRm^6^ACH’ (R = G or A; H = A, C or U) [21]. Using SRAMP and RMBase v2.0 to predict m^6^A sites, we found one m^6^A site in circRNA-SORE that is close to the junction region (Fig. 5a). It is possible that m^6^A modification plays important roles in the regulation of circRNA-SORE during the acquisition of sorafenib resistance.

In the complex mechanisms of m^6^A regulation, writers (m6A methyltransferases, such as METTL3 and METTL14) and erasers (m6A demethylases, such as FTO and ALKBH5) determine the distribution of m6A on RNA, whereas readers (such as YTHDF1 and YTHDF2) mediate m6A-dependent functions [22]. RNA pulldown assays indicated that circRNA-SORE interacted with m^6^A modifiers, such as METTL3, FTO and YTHDF1/2 (Fig. 5b). Importantly, m^6^A-specific immunoprecipitation assays showed increased m^6^A levels of circRNA-SORE in sorafenib-resistant HepG2 cells compared with wild-type controls, while m^6^A levels were reduced when METTL3 or METTL14 were silenced and increased when FTO was silenced in HepG2-SR cells (Fig. 5c-f). To further verify its existence, we designed a set of sequence-specific morpholino antisense oligos (MAOs) targeting this m^6^A site in circRNA-SORE (TGAGTCCTACCTTGATGGAGTCTCT). The m^6^A levels were significantly decreased in HepG2-SR cells treated with an MAO targeting circRNA-SORE (MAO-SORE) compared with control MAO (MAO-NC)-treated cells (Fig. 5g). Site-directed m^6^A demethylation via programmable RNA m^6^A editing by CRISPR-Cas9-ALKBH5 conjugates [23] further confirmed the existence of this m^6^A site on circRNA-SORE (Fig. 5h). Moreover, over-expression of circRNA-SORE with the m^6^A site mutated produced a lower m^6^A level than over-expression of wild-type circRNA-SORE in HepG2 cells (Fig. 5i). These results indicated that circRNA-SORE is regulated by m6A modification during the acquisition of sorafenib resistance.

m6A modification is implicated in all aspects of post-transcriptional RNA metabolism, including mRNA localization, splicing, translation and degradation, which in turn regulates important biological processes [24]. We found that the levels of circRNA-SORE were reduced by silencing adenosine methyltransferase METTL3/14 (Fig. 5j). Notably, MAO-SORE could reduce circRNA-SORE levels in sorafenib-resistant HCC cells, but not in wild-type HCC cells (Fig. 5k, l), further indicating that circRNA-SORE levels were promoted by the m^6^A modification. Intriguingly, RNA stability assays demonstrated that MAO-SORE reduced the stability of circRNA-SORE compared with MAO-NC in HepG2-SR cells (Fig. 5m), while wild-type circRNA-SORE was more stable than m^6^A-mutated circRNA-SORE under actinomycin D treatment (Fig. 5n). These results indicate that the m^6^A modification regulates circRNA-SORE expression by increasing its stability.

### Targeting circRNA-SORE in vivo retards sorafenib-resistant HCC

**Fig. 6:**
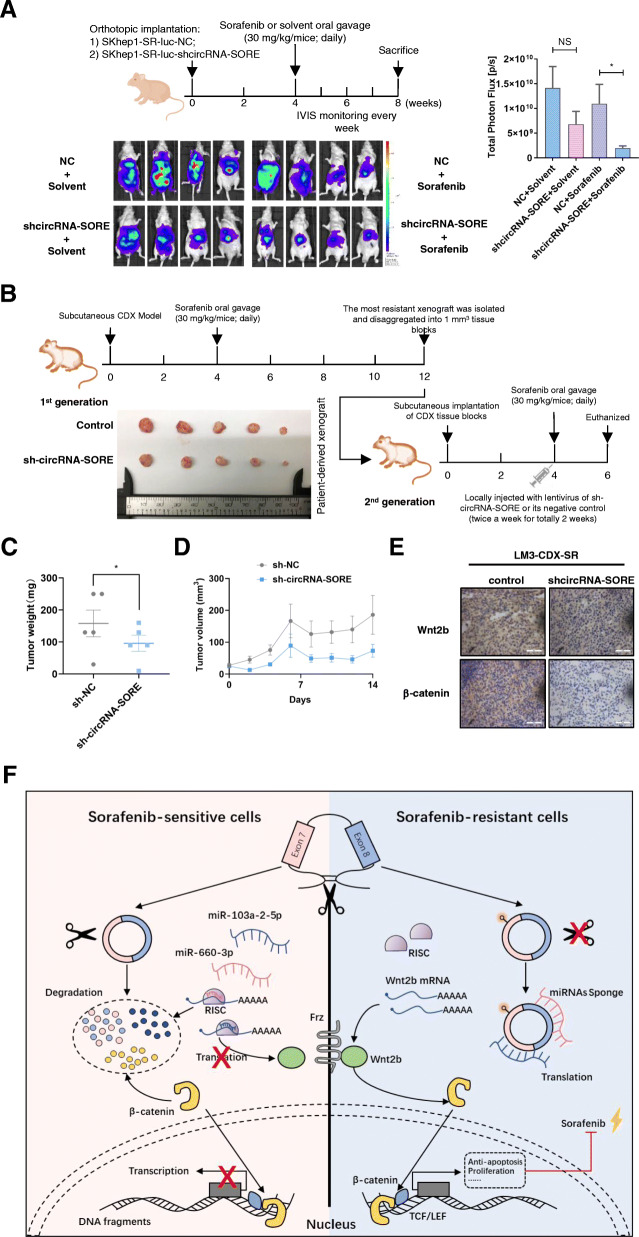
Targeting circRNA-SORE in vivo retards sorafenib-resistant HCC. **a** In vivo luminescent imaging of orthotopically implanted SKhep1-SR-luc or SKhep1-SR-luc-sh-cricRNA-SORE cells in 4–6 week-old male BALB/c nude mice treated with solvent or sorafenib (30 mg/kg/day) for 4 weeks from the fourth week after implantation. Luminescence intensity ranges from low (blue) to high (red). Tumor burdens were quantified by total photon flux (p/s). **b-d** Flow Chart of LM3-CDX-SR model construction. The first CDX generation was constructed in 4–6 week-old male BALB/c nude mice and treated with sorafenib (30 mg/kg daily, oral gavage). Twelve weeks later, the most resistant xenograft was disaggregated and implanted subcutaneously into 4–6 week-old BALB/c nude mice as the second SR-CDX. Four weeks after implantation, the second SR-CDX mice were treated with sorafenib (30 mg/kg daily, oral gavage) and locally injected with sh-circRNA-SORE lentivirus or its negative control (twice a week for 2 weeks). Mice were euthanized on the sixth week and tumors were isolated and the tumor weights were measured. The subcutaneous tumor size was measured and recorded every 2 days using the Vernier caliper as follows: tumor volume (mm^3^) = (*L* × *W*^2^)/2, where *L* is the long axis and *W* the short axis. **e** Immunohistochemistry for Wnt2b and β-catenin comparing sh-circRNA-SORE and control CDX. Scale bar, 50 μm. **f** Hypothetical model for circRNA-SORE function in HCC. circRNA-SORE acts as a sponge for miR-103a-2-5p and miR-660-3p to promote HCC sorafenib resistance by regulating the Wnt2b/β-catenin signaling pathway

To further support the in vitro findings and to discover potential clinical applications, we employed in vivo sorafenib-resistant models. Orthotopic implantations of SKhep1-SR cells expressing sh-circRNA-SORE were significantly more sensitive to sorafenib treatment than controls, while the two groups showed similar responses to solvent treatment (Fig. 6a). Moreover, local injection with sh-circRNA-SORE lentivirus around the subcutaneous implantation site in sorafenib-resistant LM3-CDX models significantly increased the sensitivity of HCC cells to sorafenib treatment (Fig. 6b-d). Similar to in vitro results, immunohistochemistry also showed impaired expression of Wnt2b and β-catenin in the sh-circRNA-SORE group (Fig. 6e).

These findings support targeting of circRNA-SORE in sorafenib-treated HCC patients as a novel pharmaceutical intervention for advanced HCC.

Overall, our results show that circRNA-SORE can sequester miR-103a-2-5p and miR-660-3p by acting as a miRNA sponge, thereby competitively activating the Wnt/β-catenin pathway and inducing sorafenib resistance. Importantly, an increased level of N6-methyladenosine (m6A) at a specific circRNA-SORE site increased RNA stability and upregulated levels of circRNA-SORE in sorafenib-resistant cells (Fig. 6f).

## Discussion

Many HCC patients are diagnosed too late for surgery because early stages of HCC present no clear symptoms. Other interventions, including molecular targeted therapy, are limited by a lack of efficacy. Unfortunately, patients who qualify for liver resection or transplantation have a high incidence of recurrence and metastases. Therefore, there is an urgent unmet medical need for the development of life-prolonging therapies. As the first US Food and Drug Administration-approved molecular targeted drug, sorafenib provided a 3 month prolongation of the median overall survival time [25, 26] and transient and limited efficacy of sorafenib was frequently reported in HCC patients. Adverse effects, such as rash, diarrhea, high blood pressure, and hand-foot syndrome, also limit high-dosage use of sorafenib [27]. Moreover, the STORM trial [28] suggested that HCC patients who accepted radical treatment (resection or ablation) could not benefit from sorafenib treatment in the adjuvant setting, posing more challenges to its clinical application. Sorafenib resistance prompts the need for new therapies to overcome resistance [29]. Multiple mechanisms underlying impaired sensitivity to sorafenib in HCC have been investigated, including Wnt/β-catenin, TGFβ, Ras/MEK/ERK, PI3K/Akt, TNFα/NF-κB, and JAK/STAT pathways, autophagy, epithelial-mesenchymal transition, cancer stem cells, tumor microenvironment, and epigenetic regulation (involving miR-222, miR-494, miR-21 and miR122) [3]. To overcome sorafenib resistance and lower its onset concentration, efforts were made to develop combined therapies [30–34]. Yet, the overall outcomes of liver cancer are still far from satisfactory. In the present study, sorafenib-resistant cell lines and animal models were developed to simulate the sorafenib resistance in HCC patients.

Non-coding RNAs are functional RNAs transcribed from the genome from which proteins cannot usually be translated. They mainly include miRNAs, long non-coding RNAs (lncRNAs) and circRNAs. Recent studies indicate that ncRNAs play vital roles in diverse biological and pathological processes, including cancer [35]. First described in 1993, miRNAs are small noncoding RNA molecules that play crucial post-transcriptional regulatory roles. Importantly, miRNAs are involved in the development of sorafenib resistance through complementary base pairing with mRNAs, predominantly in the 3′-UTR. For example, miRNA-216a/217-induced epithelial-mesenchymal transition promotes sorafenib resistance and liver cancer recurrence by targeting PTEN and SMAD7 [36]. However, its small linear structure has inherent limitations, including extensive but limited effect, poor specificity, instability, and off-target effects. In contrast to miRNAs, circRNAs have a covalently-closed loop structure with neither 5′ to 3′ polarity nor a polyadenylated tail, which makes them more stable than their linear counterparts and more resistant to RNase R degradation.

circRNAs were occasionally identified more than 20 years ago and were thought to be of low abundance and to result from alternative splicing errors during transcription. Using high-throughput sequencing and novel computational approaches, circRNAs derived from exons or introns were determined to be widespread and diverse endogenous eukaryotic ncRNAs participating in various normal and disease-related processes [37, 38]. Recent findings indicate that circRNAs function through RNA interactions, protein interactions, or by serving as transcription or splicing regulators [15]. For example, the circRNA, CDR1*as*, harbors more than 60 conserved binding sites for miR-7 [38]. Additionally, circRNAs may be associated with various miRNAs, as demonstrated for circHIPK3, which can bind to multiple miRNAs [39]. Recently, particular functions of circRNAs have been revealed in HCC; circFBLIM1 and circ-FOXP1 can act as ceRNAs to promote HCC progression [40, 41], while circMTO1 can act as a sponge of microRNA-9 to suppress HCC progression [41]. Also, study of circ_0003418 showed that circRNAs can influence anti-tumor treatments such as cisplatin [9]. Containing multiple binding sites for particular miRNAs, circRNAs have specific and efficient functionality. Furthermore, tissue- and stage-specific expression makes circRNAs potential targets for clinical intervention. Also, because of their RNA stability, circRNAs are valued as candidates for non-invasive biomarkers. circ_0005075, circ_0016788, ciRS7, circ_0128298, circ_0091579, and circ-CDYL have been identified as potential diagnostic biomarkers for HCC [42]. However, the relationship between circRNAs and resistance of HCC to sorafenib has not been reported. Thus, we aimed to fully understand the circRNA landscape when HCC patients acquired sorafenib resistance during treatment. We identified consistently up-regulated circRNA-SORE in sorafenib resistant cell lines and CDX and PDX models that was critical for the maintenance of sorafenib resistance. Importantly, orthotopic in vitro developed sorafenib-resistant CDX model and subcutaneous in vivo developed sorafenib-resistant CDX model were applied in the present study, showing that circRNA-SORE silencing could effectively reverse the acquired sorafenib resistance and retard tumor progression. In particular, we found specific mechanisms by which circRNA-SORE could specifically bind miR-103a-2-5p and miR-660-3p to act as a miRNA sponge to competitively activate Wnt/β-catenin and induce sorafenib resistance, thereby identifying a potential biomarker for prediction of sorafenib resistance and a promising therapeutic target for HCC. Derived from the 7th and 8th exons of *TLE4*, circRNA-SORE is generated into a circular form by back-splicing [43]. It was recently reported that exon-derived circRNAs are predominantly located in the cytoplasm. In our study, both nucleocytoplasmic fractionation and circRNA-FISH assays revealed that circRNA-SORE located predominantly in the cytoplasm of HCC cells.

m6A modification was first discovered in the 1970s but the development of RNA-sequencing techniques and clarification of the proteins involved make a deeper understanding of the process possible. m^6^A depends on m^6^A writers (METTL3, METTL14, KIAA1429, WTAP, RBM15 and ZC3H13), erasers (FTO and ALKBH5) and readers (YTHDC1, YTHDC2, YTHDF1, YTHDF2 and HNRNPC) [44]. m^6^A modifications may play important roles in RNA production, stability and interactions in cancers [45]. Metastasis-associated lung adenocarcinoma transcript 1 (MALAT1), a conserved lncRNA, is highly methylated with m^6^A. Two of these m^6^A residues can prevent RNA local secondary structure formation and enhance the recognition and binding of hnRNPC to a U5-tract in the MALAT1 hairpin through an “m6A switch” mechanism [46]. hnRNPA2B1 can recognize pri-miRNAs with m^6^A marks to promote interaction of DGR8 and pri-miRNAs and miRNA processing [47]. However, in HCC, the roles of m^6^A modification are intricate and controversial. Knock-down of YTHDF2 suppresses proliferation of HCC cells [48]; however, YTHDF2 was also reported to suppress HCC tumors by targeting EGFR, IL11 and SRPINE2 [49]. Indeed, the detailed roles of m^6^A modification in HCC need further investigation. Recently, m^6^A-modified circRNAs were found with cell-type-specific expression [45]. m^6^A can recruit YTHDF3 and initiation factor, eIF4G2, to regulate protein synthesis from circRNAs [19]. The change of m^6^A level by ALKBH5 and METTL3 affects circRNA biosynthesis in spermatogenesis by regulating enhanced splicing and promotes circRNA formation [50]. YTHDF2–HRSP12–RNase P/MRP-mediated endoribonucleolytic cleavage is related to m^6^A-containing circRNA decay [51]. However, the functions of m^6^A-modified circRNAs in HCC and sorafenib resistance were still elusive.

In this study, we identified a predicted m^6^A site in circRNA-SORE by a series of experiments. Our results show that the m^6^A level of circRNA-SORE is increased in sorafenib-resistant cells, and that the expression of circRNA-SORE is decreased when its m^6^A modification was inhibited. Recently, lncRNA GAS5-AS was found to enhance GAS5 stability by interacting with ALKBH5 and regulating m^6^A modifications of GAS5, which was dependent on ALKBH5 and YTHDF2 [52]. In the present study, mechanistic dissection suggested that the m^6^A modification can stabilize circRNA-SORE. However, no significant differences in the expression level of m^6^A-related proteins were found between sorafenib-resistant and parental cells. The mechanism of increased circRNA-SORE m^6^A levels in sorafenib-resistant cells requires further investigation.

## Conclusion

To conclude, upregulated circRNA-SORE was necessary for the maintenance of sorafenib resistance, and silencing circRNA-SORE substantially increased the efficacy of sorafenib-induced apoptosis. Mechanistic studies revealed that circRNA-SORE sequestered miR-103a-2-5p and miR-660-3p by acting as a miRNA sponge, thereby competitively activating the Wnt/β-catenin pathway and inducing sorafenib resistance. Importantly, the increased levels of circRNA-SORE in sorafenib-resistant cells resulted from increased RNA stability, which was triggered by increased levels of m^6^A at specific sites in circRNA-SORE. In vivo delivery of circRNA-SORE RNAi by local short hairpin RNA lentivirus injection substantially enhanced sorafenib efficacy in animal models. This work describes a novel mechanism of sorafenib resistance maintenance and is a proof-of-concept demonstration for targeting circRNA-SORE in sorafenib-treated HCC patients as a novel pharmaceutical intervention for advanced HCC.

## Supplementary Information

**Additional file 1.**

## Data Availability

All data generated or analyzed during this study are included either in this article or in the supplementary information files.

## Abbreviations

HCC: Hepatocellular carcinoma
SHARP: Sorafenib Hepatocellular Carcinoma Assessment Randomized Protocol
circRNAs: Circular RNAs
ncRNAs: non-coding RNAs
AS: Alternative splicing
ceRNAs: competing endogenous RNAs
m^6^A: N6-methyladenosine
ALL: Acute lymphoblastic leukemia
HBXIP: Hepatitis B X-interacting protein
miRNA: microRNA
a circular RNA up-regulated in sorafenib-resistant HCC cells: circRNA-SORE
SR: Sorafenib-resistant
CDX: Cell-derived xenograft
PDX: Patient-derived xenograft
FISH: Fluorescence in situ hybridization
RISC: RNA-induced silencing complex
TCGA: The Cancer Genome Atlas
3′-UTR: 3′-untranslated region
lncRNAs: long non-coding RNAs
MALAT1: Metastasis-associated lung adenocarcinoma transcript 1

## References

[1] Forner A, Reig M, Bruix J (2018). Hepatocellular carcinoma. Lancet.

[2] Connell LC, Harding JJ, Abou-Alfa GK (2016). Advanced hepatocellular Cancer: the current state of future research. Curr Treat Options in Oncol.

[3] Xia S, Pan Y, Liang Y, Xu J, Cai X (2020). The microenvironmental and metabolic aspects of sorafenib resistance in hepatocellular carcinoma. EBioMedicine.

[4] Lasda E, Parker R (2014). Circular RNAs: diversity of form and function. RNA (New York).

[5] Zhang L, Zhou Q, Qiu Q, Hou L, Wu M, Li J, Li X, Lu B, Cheng X, Liu P (2019). CircPLEKHM3 acts as a tumor suppressor through regulation of the miR-9/BRCA1/DNAJB6/KLF4/AKT1 axis in ovarian cancer. Mol Cancer.

[6] Li Z, Ruan Y, Zhang H, Shen Y, Li T, Xiao B. Tumor-suppressive circular RNAs: mechanisms underlying their suppression of tumor occurrence and use as therapeutic targets. Cancer Sci. 2019;110(12):3630-8. 10.1111/cas.14211 PMC6890437 31599076

[7] Su Y, Lv X, Yin W, Zhou L, Hu Y, Zhou A, Qi F (2019). CircRNA Cdr1as functions as a competitive endogenous RNA to promote hepatocellular carcinoma progression. Aging.

[8] Wang L, Long H, Zheng Q, Bo X, Xiao X, Li B (2019). Circular RNA circRHOT1 promotes hepatocellular carcinoma progression by initiation of NR2F6 expression. Mol Cancer.

[9] Chen H, Liu S, Li M, Huang P, Li X (2019). circ_0003418 inhibits tumorigenesis and Cisplatin Chemoresistance through Wnt/β-catenin pathway in hepatocellular carcinoma. OncoTargets Ther.

[10] Lee Y, Choe J, Park OH, Kim YK (2020). Molecular mechanisms driving mRNA degradation by m (6) a modification. Trends Genet.

[11] Choe J, Lin S, Zhang W, Liu Q, Wang L, Ramirez-Moya J, Du P, Kim W, Tang S, Sliz P (2018). mRNA circularization by METTL3-eIF3h enhances translation and promotes oncogenesis. Nature.

[12] Vu LP, Pickering BF, Cheng Y, Zaccara S, Nguyen D, Minuesa G, Chou T, Chow A, Saletore Y, MacKay M (2017). The N (6)-methyladenosine (m (6) a)-forming enzyme METTL3 controls myeloid differentiation of normal hematopoietic and leukemia cells. Nat Med.

[13] Cai X, Wang X, Cao C, Gao Y, Zhang S, Yang Z, Liu Y, Zhang X, Zhang W, Ye L (2018). HBXIP-elevated methyltransferase METTL3 promotes the progression of breast cancer via inhibiting tumor suppressor let-7g. Cancer Lett.

[14] Liu X, Liu J, Xiao W, Zeng Q, Bo H, Zhu Y, Gong L, He D, Xing X, Li R, et al. SIRT1 regulates N (6) -methyladenosine RNA modification in hepatocarcinogenesis by inducing RANBP2-dependent FTO SUMOylation. Hepatology. 2020. 10.1002/hep.31222 32154934

[15] Lyu D, Huang S. The emerging role and clinical implication of human exonic circular RNA. RNA Biol. 2017;14(8):1000-6. 10.1080/15476286.2016.1227904 PMC5680672 27588461

[16] Enright AJ, John B, Gaul U, Tuschl T, Sander C, Marks DS (2003). MicroRNA targets in drosophila. Genome Biol.

[17] Pasquinelli AE (2012). MicroRNAs and their targets: recognition, regulation and an emerging reciprocal relationship. Nat Rev Genet.

[18] Tang Z, Li C, Kang B, Gao G, Li C, Zhang Z. GEPIA: a web server for cancer and normal gene expression profiling and interactive analyses. Nucleic Acids Res. 2017;45(W1):W98-102. 10.1093/nar/gkx247 PMC5570223 28407145

[19] Yang Y, Fan X, Mao M, Song X, Wu P, Zhang Y, Jin Y, Yang Y, Chen LL, Wang Y (2017). Extensive translation of circular RNAs driven by N (6)-methyladenosine. Cell Res.

[20] Chen RX, Chen X, Xia LP, Zhang JX, Pan ZZ, Ma XD, Han K, Chen JW, Judde JG, Deas O (2019). N (6)-methyladenosine modification of circNSUN2 facilitates cytoplasmic export and stabilizes HMGA2 to promote colorectal liver metastasis. Nat Commun.

[21] Csepany T, Lin A, Baldick CJ, Beemon K (1990). Sequence specificity of mRNA N6-adenosine methyltransferase. J Biol Chem.

[22] Fu Y, Dominissini D, Rechavi G, He C (2014). Gene expression regulation mediated through reversible m (6) a RNA methylation. Nat Rev Genet.

[23] Liu XM, Zhou J, Mao Y, Ji Q, Qian SB (2019). Programmable RNA N (6)-methyladenosine editing by CRISPR-Cas9 conjugates. Nat Chem Biol.

[24] Meyer KD, Jaffrey SR (2014). The dynamic epitranscriptome: N6-methyladenosine and gene expression control. Nat Rev Mol Cell Biol.

[25] Cheng AL, Kang YK, Chen Z, Tsao CJ, Qin S, Kim JS, Luo R, Feng J, Ye S, Yang TS (2009). Efficacy and safety of sorafenib in patients in the Asia-Pacific region with advanced hepatocellular carcinoma: a phase III randomised, double-blind, placebo-controlled trial. Lancet Oncol.

[26] Llovet JM, Ricci S, Mazzaferro V, Hilgard P, Gane E, Blanc JF, de Oliveira AC, Santoro A, Raoul JL, Forner A (2008). Sorafenib in advanced hepatocellular carcinoma. N Engl J Med.

[27] Hampton T (2007). Cancer drug trials show modest benefit: drugs target liver, gastric, head and neck cancers. JAMA.

[28] Bruix J, Takayama T, Mazzaferro V, Chau GY, Yang J, Kudo M, Cai J, Poon RT, Han KH, Tak WY (2015). Adjuvant sorafenib for hepatocellular carcinoma after resection or ablation (STORM): a phase 3, randomised, double-blind, placebo-controlled trial. Lancet Oncol.

[29] Chen J, Jin R, Zhao J, Liu J, Ying H, Yan H, Zhou S, Liang Y, Huang D, Liang X (2015). Potential molecular, cellular and microenvironmental mechanism of sorafenib resistance in hepatocellular carcinoma. Cancer Lett.

[30] Xu J, Zheng L, Chen J, Sun Y, Lin H, Jin R-A, Tang M, Liang X, Cai X (2017). Increasing AR by HIF-2α inhibitor (PT-2385) overcomes the side-effects of sorafenib by suppressing hepatocellular carcinoma invasion via alteration of pSTAT3, pAKT and pERK signals. Cell Death Dis.

[31] Ji L, Lin Z, Wan Z, Xia S, Jiang S, Cen D, Cai L, Xu J, Cai X (2020). miR-486-3p mediates hepatocellular carcinoma sorafenib resistance by targeting FGFR4 and EGFR. Cell Death Dis.

[32] Lin Z, Xia S, Liang Y, Ji L, Pan Y, Jiang S, Wan Z, Tao L, Chen J, Lin C (2020). LXR activation potentiates sorafenib sensitivity in HCC by activating microRNA-378a transcription. Theranostics.

[33] Xu J, Lin H, Li G, Sun Y, Chen J, Shi L, Cai X, Chang C (2016). The miR-367-3p increases Sorafenib chemotherapy efficacy to suppress hepatocellular carcinoma metastasis through altering the androgen receptor signals. EBioMedicine.

[34] Xu J, Lin H, Li G, Sun Y, Shi L, Ma WL, Chen J, Cai X, Chang C (2017). Sorafenib with ASC-J9((R)) synergistically suppresses the HCC progression via altering the pSTAT3-CCL2/Bcl2 signals. Int J Cancer.

[35] Anastasiadou E, Jacob LS, Slack FJ (2018). Non-coding RNA networks in cancer. Nat Rev Cancer.

[36] Xia H, Ooi LL, Hui KM (2013). MicroRNA-216a/217-induced epithelial-mesenchymal transition targets PTEN and SMAD7 to promote drug resistance and recurrence of liver cancer. Hepatology.

[37] Memczak S, Jens M, Elefsinioti A, Torti F, Krueger J, Rybak A, Maier L, Mackowiak SD, Gregersen LH, Munschauer M (2013). Circular RNAs are a large class of animal RNAs with regulatory potency. Nature.

[38] Hansen TB, Jensen TI, Clausen BH, Bramsen JB, Finsen B, Damgaard CK, Kjems J (2013). Natural RNA circles function as efficient microRNA sponges. Nature.

[39] Zheng Q, Bao C, Guo W, Li S, Chen J, Chen B, Luo Y, Lyu D, Li Y, Shi G (2016). Circular RNA profiling reveals an abundant circHIPK3 that regulates cell growth by sponging multiple miRNAs. Nat Commun.

[40] Bai N, Peng E, Qiu X, Lyu N, Zhang Z, Tao Y, Li X, Wang Z (2018). circFBLIM1 act as a ceRNA to promote hepatocellular cancer progression by sponging miR-346. J Ex Clin Cancer Res.

[41] Han D, Li J, Wang H, Su X, Hou J, Gu Y, Qian C, Lin Y, Liu X, Huang M (2017). Circular RNA circMTO1 acts as the sponge of microRNA-9 to suppress hepatocellular carcinoma progression. Hepatology.

[42] Qiu L, Xu H, Ji M, Shang D, Lu Z, Wu Y, Tu Z, Liu H (2019). Circular RNAs in hepatocellular carcinoma: biomarkers, functions and mechanisms. Life Sci.

[43] Hentze MW, Preiss T (2013). Circular RNAs: splicing's enigma variations. EMBO J.

[44] Yang Y, Hsu PJ, Chen YS, Yang YG (2018). Dynamic transcriptomic m (6) a decoration: writers, erasers, readers and functions in RNA metabolism. Cell Res.

[45] Zhou C, Molinie B, Daneshvar K, Pondick JV, Wang J, Van Wittenberghe N, Xing Y, Giallourakis CC, Mullen AC (2017). Genome-wide maps of m6A circRNAs identify widespread and cell-type-specific methylation patterns that are distinct from mRNAs. Cell Rep.

[46] Liu N, Dai Q, Zheng G, He C, Parisien M, Pan T (2015). N (6)-methyladenosine-dependent RNA structural switches regulate RNA-protein interactions. Nature.

[47] Alarcón CR, Goodarzi H, Lee H, Liu X, Tavazoie S, Tavazoie SF (2015). HNRNPA2B1 is a mediator of m (6) A-dependent nuclear RNA processing events. Cell.

[48] Yang Z, Li J, Feng G, Gao S, Wang Y, Zhang S, Liu Y, Ye L, Li Y, Zhang X (2017). MicroRNA-145 modulates N (6)-Methyladenosine levels by targeting the 3′-Untranslated mRNA region of the N (6)-Methyladenosine binding YTH domain family 2 protein. J Biol Chem.

[49] Zhong L, Liao D, Zhang M, Zeng C, Li X, Zhang R, Ma H, Kang T (2019). YTHDF2 suppresses cell proliferation and growth via destabilizing the EGFR mRNA in hepatocellular carcinoma. Cancer Lett.

[50] Tang C, Xie Y, Yu T, Liu N, Wang Z, Woolsey RJ, Tang Y, Zhang X, Qin W, Zhang Y (2020). m (6) A-dependent biogenesis of circular RNAs in male germ cells. Cell Res.

[51] Park OH, Ha H, Lee Y, Boo SH, Kwon DH, Song HK, Kim YK (2019). Endoribonucleolytic Cleavage of m (6) A-Containing RNAs by RNase P/MRP Complex. Mol Cell.

[52] Wang X, Zhang J, Wang Y (2019). Long noncoding RNA GAS5-AS1 suppresses growth and metastasis of cervical cancer by increasing GAS5 stability. Am J Transl Res.

