# Supplementary material for: N6-methyladenosine-modified CircRNA-SORE sustains sorafenib resistance in hepatocellular carcinoma by regulating β-catenin signaling
Source: Mol Cancer. 2020 Nov 23;19:163. doi: 10.1186/s12943-020-01281-8 (PMC7681956; doi:10.1186/s12943-020-01281-8)
Supplement: Supplementary file 1 — Additional file 1. [file 12943_2020_1281_MOESM1_ESM.docx]

**Supplementary Materials**

**Materials:** Sorafenib was purchased from Selleck Chemicals (Houston, TX, USA). Actinomycin D was purchased from Sigma-Aldrich (St. Louis, MO, USA). RNase R was purchased from Epicentre (Madison, WI, USA). Cycloheximide (CHX) was purchased from MedChemExpress (Monmouth Junction, NJ, USA).

***In vitro* cell culture/maintenance:** The human HCC cell line HepG2 (RRID: CVCL_0027) and SKhep1 (RRID: CVCL_0525) were purchased from the American Type Culture Collection (ATCC, Manassas, VA, USA). Huh7 and LM3 were purchased from the China Center for Type Culture (CCTCC, Wuhan, Hubei, China). All the cell lines were mycoplasma negative and identified with authentication reports. Mycoplasma Test Kit GMyc-PCR (YEASEN, Shanghai, China) was used for mycoplasma detection. All the cell lines were cultured in Dulbecco's Modified Eagle's Media (Invitrogen, Grand Island, NY, USA) supplemented with 10% FBS (v/v), penicillin (25 units/ml), streptomycin (25 g/ml), 1% L-glutamine, and 10% fetal bovine serum (FBS) in a 5% (v/v) CO_2_ humidified incubator at 37°C. CCK-8 assays were conducted to confirm the resistance of each cell line to sorafenib. Cell growth curves were automatically recorded on the xCELLigence System (Roche Applied Sciences) in real-time. The cell index was followed for 3 days. For treatment with MAOs, each MAO (GeneTools, Philomath, OR, USA) was added to culture media at a final concentration of 10 μM along with Endoporter (GeneTools, Philomath, OR, USA) and incubated for 24 hours. The sequence of MAOs targeting the predicted m^6^A site on circRNA-SORE: TGAGTCCTACCTTGATGGAGTCTCT.

**Western blot analysis:** Cells were lysed in lysis buffer and proteins (30 µg) were separated on 10–12% SDS/PAGE gel and then transferred onto PVDF membranes (Millipore, Billerica, MA). After blocking membranes, they were incubated with appropriate dilutions of specific primary antibodies against β-tubulin, wnt2b, Ago2 (from Abcam, Cambridge, MA) and β-catenin (from Cell Signaling, Danvers, MA). The blots were incubated with HRP-conjugated secondary antibodies and visualized using the ECL system (Thermo Fisher Scientific, Rochester, NY).

**CCK8 assay and colony formation assay:** The viability of HCC cells was determined by Cell Counting Kit 8 (Dojindo, Japan) and measured at OD 450 nm with the BioTek Gen5 system (BioTeck, USA). 6-cm dishes were seeded with 1 × 10^4^ viable cells and allowed to grow for 24 hours. The cells were then incubated in the presence or absence of designated siRNA transfection for 24 hours in complete media, then washed with PBS gently and allowed to grow in complete media for another 7 days with sorafenib treatment. The colonies obtained were then washed gently with PBS and fixed in 4% paraformaldehyde for 20 minutes at room temperature and then washed with PBS followed by staining with 0.1% (w/v) crystal violet. The colonies with >50 cells under microscope were counted. Three different independent experiments were performed.

**RNA extraction, miRNA extraction, and reverse transcription and Quantitative Real-Time PCR analysis:** For RNA extraction, total RNA was isolated using Trizol reagent (Invitrogen, Grand Island, NY). 1 µg of total RNA was subjected to reverse transcription using Superscript III transcriptase (Invitrogen, Grand Island, NY). Quantitative real-time PCR (qRT-PCR) was conducted using a Bio-Rad CFX96 system with SYBR green to determine the mRNA expression level of a gene of interest. Expression levels were normalized to the expression of β-actin RNA. miRNA was isolated by using PureLink® miRNA kit. Briefly, 50 ng of miRNA was processed for poly A addition by adding 1 unit of polymerase with 1 mM ATP in 1x RT buffer at 37℃ for 10 minutes in 10 μl volume. The sample was heat inactivated at 95℃ for 2 minutes. 50 of pmol anchor primer was added to 12.5 μl, incubated at 65℃ for 5 minutes. For the last step of cDNA synthesis, we added 2 μl of 5x RT buffer, 2 μl of 10mM dNTP, and 1 μl of reverse transcriptase for a total 20μl. The sample was then incubated at 42 ℃ for 1 hour. Quantitative real-time PCR (qRT-PCR) was conducted using a Bio-Rad CFX96 system with SYBR green to determine the mRNA expression level of the gene of interest. Expression levels were normalized to the expression of 5s RNA.

**Sequences of primers in this study**

| circRNA-SORE | Forward | GTGCTCTAGGAGGTCAGTCC |
| --- | --- | --- |
|  | Reverse | GTGTGGAGTCAGAGGTACGG |
|  | Divergent Forward | ACAGAGACTCCATCAAG |
|  | Divergent Reverse | TTTAATTGGAAGATGGGACT |
|  | Convergent Forward | GTCCCATCTTCCAATTAAA |
|  | Convergent Reverse | CTTGATGGAGTCTCTGT |
|  | Divergent Forward  (Amplification for Sequencing) | CCATGACAATGATCACCAA |
|  | Divergent Reverse  (Amplification and Sequencing) | TTTAATTGGAAGATGGGAC |
| TLE4 | Forward | GCAGAGATTGTCAAGAGGCTG |
|  | Reverse | GTGATAAATGCTGGGCCTGG |
| GAPDH | Forward | CACATCGCTCAGACACCATG |
|  | Reverse | TTGAGGTCAATGAAGGGGTC |
| SLUG | Forward | AGACCCCCATGCCATTGAAG |
|  | Reverse | CTTCTCCCCCGTGTGAGTTC |
| TWIST | Forward | CCCGTGGACAGTGATTCCC |
|  | Reverse | CTTTCAGTGGCTGATTGGCAC |
| PPAR Delta | Forward | GAGAATTCTGCGGAGCCTGC |
|  | Reverse | ACCATTCCAGACCCTCGTTG |
| CD44 | Forward | CACACCCTCCCCTCATTCAC |
|  | Reverse | TGGATGGCTGGTATGAGCTG |
| AXIN2 | Forward | GGGATCACTGGCTCCGC |
|  | Reverse | TCCATCGCTCTGAGGGGTTA |
| MYC | Forward | GCAATGCGTTGCTGGGTTAT |
|  | Reverse | CGCATCCTTGTCCTGTGAGT |
| TIAM1 | Forward | CAGGGCAGAGACGCTTACAT |
|  | Reverse | TCTGGGAAGCTGTCAAAGGC |
| MMP7 | Forward | CAGGAAACACGCTGGCTCAT |
|  | Reverse | CCCTAGACTGCTACCATCCG |
| m^6^A site on circRNA-SORE | Forward | AGCACCATGACAATGATCACCA |
|  | Reverse | TACCGATGGGTGGAATGGC |
| miRNA-9-5p | Forward | CGGTCTTTGGTTATCTAGCTGTATG |
| miRNA-96-3p | Forward | GGAATCATGTGCAGTGCCAATA |
| miRNA-103a-2-5p | Forward | AGCTTCTTTACAGTGCTGCCTTG |
| miRNA-149-3p | Forward | TAAGGGAGGGACGGGGGCTG |
| miRNA-185-3p | Forward | AGGGGCTGGCTTTCCTCTGGTC |
| miRNA-328-5p | Forward | ATAATGGGGGGGCAGGAG |
| miRNA-345-3p | Forward | GCCCTGAACGAGGGGTCTGGAG |
| miRNA-363-5p | Forward | CGGGTGGATCACGATGCA |
| miRNA-591 | Forward | GGCGAGACCATGGGTTCTCATTGT |
| miRNA-609 | Forward | GGCGCAGGGTGTTTCTCTCATCTC |
| miRNA-660-3p | Forward | CACCTCCTGTGTGCATGGATT |
| miRNA-762 | Forward | ATTATGGGGCTGGGGCCGGGGC |
| 5S | Forward | GGGAATACCGGGTGCTGTAGGCT |

**Programmable RNA N(6)-methyladenosine editing by CRISPR-Cas9 conjugates:** ALKBH5-dCas9 plasmids were obtained from Gene Script (ALKBH5-dCas9). Briefly, Full-length coding sequence of human ALKBH5 was directly cloned into pLenti_dCas9-VP64_Blast vector using CloneEZ to generate ALKBH5-dCas9. Four different sgRNAs were designed based on the target m6A site specific for circRNA-SORE and cloned into gRNA_pGS-gRNA-Neo vector. The sgRNAs sequences: sgRNA 1: 5‘-GCCCTCTCCAGTGCTCTAGG; sgRNA 2: GCCCTCTCCAGTGCTCTAGG; sgRNA 3: TGATGGAGTCTCTGTCTCTT; sgRNA 4: TGATGGAGTCTCTGTCTCTT. ALKBH5-dCas9 and its vehicle retroviral constructs were cotransfected with PAX2 and VSVG constructs into 293 cells in DMEM medium using Lipofectamine 2000(Invitrogen). The medium was then filtered and used for transduction in HepG2-SR cell line. Cells were selected in 10 ug/ml blasticidin for 5 days. After selection, cells were transfected with sgRNAs using Lipofectamine 3000(Invitrogen). Cells were selected in 2 ug/ml neomycin for 5 days.

**Luciferase reporter assay:** The wildtype and mutant 3’ UTR of wnt2B was amplified and subcloned into the pGL3-Basic backbone, and the wildtype and mutant linear form of circRNA-SORE was amplified and subcloned into the psiCheck2 backbone (Detailed sequence see **Table S3 and Table S4**). For the luciferase assay, cells were plated in 24-well plates and the cDNA transfected using Lipofectamine 3000 (Invitrogen) according to the manufacturer's instruction. pRL-TK was used as internal control in company with pGL3-Basic. Luciferase activity was measured by Dual-Luciferase Assay (Promega) according to the manufacturer's manual and Renilla luciferase activity was normalized against Firefly luciferase activity.

***In vivo* studies:** For our orthotopic xenograft mice model, 24 4- to 6-week-old male athymic BALB/c nude mice (SLAC- Shanghai Laboratory Animal Center, China) were housed and fed in standard pathogen-free conditions. Sorafenib-resistant SKhep1 cells (SKhep1-SR) were prepared as stable luciferase clones by transduction with pLKO-Luciferase lentivirus and GV248 or GV248-shcircRNA-SORE, and were then selected with G418 and puromycin to expand in culture. Intrahepatic injections of 5 × 10^6^ cells/100μL serum-free DMEM and Matrigel (1:1) were performed on each nude mouse in groups (6 mice/group) as follows: 1) SKhep1-SR-luc-NC + Solvent; 2) SKhep1-SR-luc-NC + Sorafenib; 3) SKhep1-SR-luc-NC + Solvent; 4) SKhep1-SR-luc-shcircRNA-SORE + Sorafenib. All surgeries were performed under sodium pentobarbital anesthesia, and all efforts were made to minimize suffering. Sorafenib was suspended in an oral vehicle containing Cremophor (Sigma-Aldrich), 95% ethanol and water in a ratio of 1:1:6. Four weeks later, the mice were treated with solvent or sorafenib (30 mg/kg/mouse; daily, oral gavage) as indicated for another month. Tumor development was monitored by IVIS once a week starting from drug treatment following intraperitoneal injection of 150mg/kg D-Luciferin. Total photon flux was measured using the software. Mice were sacrificed after 4 weeks of treatment.

For our subcutaneous cell derived xenograft (CDX) model, subcutaneous injections of 5 × 10^6^ LM3 cells/100μL serum-free DMEM and Matrigel (1:1) were performed into the axilla of 4–6 week-old male BALB/c nude mice (SLAC-Shanghai Laboratory Animal Center, China). When the xenografts reached approximately 100 mm^3^ (approximately four weeks), all the mice were treated with sorafenib (30 mg/kg/mice; daily, oral gavage). 12 weeks later, the most resistant xenografts were isolated and mechanically disaggregated into approximately 1 mm^3^ tissue blocks to sub-transplant into the axilla of 4-6 week-old male BALB/c nude mice for the second CDX generation. When the xenografts reached approximately 100 mm^3^ (approximately four weeks), the mice were randomized into two groups (5 mice in each group): 1) negative control and 2) sh-circRNA-SORE. All the mice were treated with sorafenib (30 mg/kg/mouse; daily, oral gavage) and each tumor was locally injected with sh-circRNA-SORE lentivirus or its negative control twice a week for two weeks. Mice were euthanized on the sixth week and tumors were isolated for further studies.

For our PDX models, the fragments of fresh human HCC tissues were transplanted subcutaneously into the axilla of 4-6 week-old NOD/SCID mice (SLAC-Shanghai Laboratory Animal Center, China). When the xenografts reached approximately 100 mm^3^ (approximately four weeks), all the mice were treated with sorafenib (30 mg/kg/mice; daily, oral gavage). 12 weeks later, the mice were euthanized and tumors were isolated for further studies.

The subcutaneous tumor size was measured and recorded every 2 days using the Vernier caliper as follows: tumor volume (mm^3^) = (*L*×*W*^2^)/2, where *L* is the long axis and *W* the short axis. All animal experiments were performed humanely in compliance with guidelines reviewed by the Animal Ethics Committee of the Biological Resource Centre of the Agency for Science, Technology and Research at the Sir Run-Run Shaw Hospital.

**H&E and immunohistochemical (IHC) staining**: Tissues were fixed in 10% (v/v) formaldehyde in PBS, embedded in paraffin, and cut into 5 μm sections and used for H&E staining and IHC staining with specific primary antibodies against wnt2b (Abcam, Cambridge, MA), and β-catenin (Cell Signaling, Danvers, MA). To enhance antigen exposure, the slides were treated with 1 × EDTA at 98°C for 10 minutes for antigen retrieval. The slides were incubated with endogenous peroxidase blocking solution, and then were incubated with the primary antibody at 4 ℃ overnight. After rinsing with Tris-buffered saline, the slides were incubated for 45 minutes with biotin-conjugated secondary antibody, washed, and then incubated with enzyme conjugate horseradish peroxidase (HRP)-streptavidin. Freshly prepared DAB (Zymed, South San Francisco, CA) was used as substrate to detect HRP. Finally, slides were counter-stained with hematoxylin and mounted with aqueous mounting media. Positive cells were calculated as the number of immunopositive cells × 100% divided by total number of cells/field in 10 random fields at 400 × magnification. The slides were reviewed and scored by an experienced pathologist without the knowledge of patient outcome. The staining results were measured semiquantitatively.

**Immunofluorescence (IF) and RNA Fluorescence in situ hybridization (FISH)：**Immunofluorescence was performed using specific antibody to YBX1. HepG2-SR were fixed with 4% formaldehyde (Fisher) for 15 min and then blocked with 5% normal goat serum (Vector) with or without 0.1% Triton X-100 in PBS for 60 minutes at room temperature. Immunostaining was performed using the appropriate primary and secondary antibodies. Nuclei were counterstained with DAPI.

In situ hybridization was performed using specific probes to circRNA-SORE sequence. Digoxin-labelled RNA probes were transcribed from PCR fragments using the DIG RNA labelling mix and T7 RNA polymerase (Roche) according to the manufacturers’ instructions. HepG2-SR were grown to the exponential phase and were 80–95% confluent at the time of fixation. After prehybridization (1×PBS/0.5% Triton X-100), cells were hybridized in hybridization buffer (40% formamide, 10% Dextran sulfate, 1×Denhardt’s solution, 4×SSC, 10mM DDT, 1mg ml^-1^ yeast transfer RNA, 1mg ml^-1^ sheared salmon sperm DNA) with DIG-labelled probes specific to circRNA-SORE at 60℃ overnight and subsequently with anti-DIG-FITC at 37℃ for 1 hour. Nuclei were counterstained with 4,6-diamidino-2-phenylindole (DAPI). The images were acquired on a TCS SP2 AOBS confocal microscope.

**RNA Immunoprecipitation (RIP) and Methylated RNA Immunoprecipitation (MeRIP):** RNA immunoprecipitation was performed with the Protein A/G Agarose Beads (Santa Cruz) according to the manufacturer’s instructions. Briefly, Protein A/G Agarose Beads coated with 5 mg of normal antibodies against rabbit immunoglobulin G (Beyotime), Ago2 (Abcam), or m^6^A (Abcam) were incubated with pre-frozen cell lysates or nuclear extracts overnight at 4℃. Associated RNA-protein complexes were collected and washed 6 times and then subjected to proteinase K digestion and RNA extraction by TRIzol. The relative interaction between protein and RNA was determined by qPCR, PCR, and normalized to input.

**JC-1 assay:** Following the designated treatments, all cells including both floating and attached cells were collected by trypsinization (0.25% Trypsin, without EDTA (Gibco) and washed with PBS. The apoptotic cells were detected by Annexin V-FITC Apoptosis Detection Kit I (BD Biosciences) by staining with Annexin V-FITC and PI according to the supplier’s instructions. Viable and dead cells were detected by a BD LSRII flow cytometer (BD Biosciences).

**Statistical Analysis:** Data are expressed as mean ± SEM from at least three independent experiments. Statistical analyses used Student’s t-test, Kaplan-Meier survival analysis and log-rank test with GraphPad Prism 5 (GraphPad Software, Inc., La Jolla, CA). P <0.05 was considered statistically significant. Gene Ontology Analysis was performed online by Database for Annotation, Visualization and Integrated Discovery (DAVID) bioinformatics resources (v6.8)^[1](#_ENREF_1), [2](#_ENREF_2)^.

References:

1. Huang da W, Sherman BT, Lempicki RA. Systematic and integrative analysis of large gene lists using DAVID bioinformatics resources. Nat Protoc 2009;4:44-57.

2. Huang da W, Sherman BT, Lempicki RA. Bioinformatics enrichment tools: paths toward the comprehensive functional analysis of large gene lists. Nucleic Acids Res 2009;37:1-13.

3. Kanehisa M, Sato Y, Kawashima M, et al. KEGG as a reference resource for gene and protein annotation. Nucleic Acids Res 2016;44:D457-62.

**Table S1. Sequences of siRNAs against specific target in this study**

| circRNA-SORE siRNA-1 | Sense (5’-3’) | ACUCCAUCAAGCAGCAACAdTdT |
| --- | --- | --- |
| circRNA-SORE siRNA-2 | Sense (5’-3’) | CAUCAAGCAGCAACAACUCdTdT |
| β-catenin siRNA | Sense (5’-3’) | AGCUGAUAUUGAUGGACAGdTdT |
| METTL3 siRNA | Sense (5’-3’) | GCUGCACUUCAGACGAAUUAUdTdT |
| METTL14 siRNA | Sense (5’-3’) | GCUUACAAAUAGCAACUACAAdTdT |
| FTO siRNA | Sense (5’-3’) | CCCAUUAGGUGCCCAUAUUUAdTdT |

**Table S2. The sequence of wildtype and mutant of circRNA-SORE**

**Wildtype of circRNA-SORE:**

CAGCAACAACTCCAGGCCCAGCATTTATCACATGGACATGGTCTCCCCGTACCTCTGACTCCACACCCTTCAGGGCTCCAGCCCCCTGCCATTCCACCCATCGGTAGCAGTGCCGGGCTTCTGGCCCTCTCCAGTGCTCTAGGAGGTCAGTCCCATCTTCCAATTAAAGATGAGAAGAAGCACCATGACAATGATCACCAAAGAGACAG**AGACT**CCATCAAG

Bold: the wildtype m^6^A site on circRNA-SORE

**Mutation** **of circRNA-SORE**:

CAGCAACAACTCCAGGCCCAGCATTTATCACATGGACATGGTCTCCCCGTACCTCTGACTCCACACCCTTCAGGGCTCCAGCCCCCTGCCATTCCACCCATCGGTAGCAGTGCCGGGCTTCTGGCCCTCTCCAGTGCTCTAGGAGGTCAGTCCCATCTTCCAATTAAAGATGAGAAGAAGCACCATGACAATGATCACCAAAGAGACAG**CGCCG**CCATCAAG

Bold: the mutant m^6^A site on circRNA-SORE

**Table S3. The sequence of wildtype and mutant 3’ UTR of WNT2B and linear form of circRNA-SORE**

**Wildtype 3’ UTR of WNT2B:**

ACACACAGATACCTCACTCATCCCTCCAATTCAAGCCTCTCAACTCAAAAGCACAAGATCCTTGCATGCACACCTTCCTCCACCCTCCACCCTGGGCTGCTACCGCTTCTATTTAAGGATGTAGAGAGTAATCCATAGGGACCATGGTGTCCTGGCTGGTTCCTTAGCCCTGGGAAGGAGTTGTCAGGGGATATAAGAAACTGAGCAAGCTCCCTGATTTCCCGCTCTGGAGATTTGAAGGGAGAGTAGAAGAGATAGGGGGTCTTTAGAGTGAAATGAGTTGCACTAAAGTACGTAGTTGAGGCTCCTTTTTTCTTTCCTTTGCACCAGCTTCCCGATACTTCTTGGTGTGCAAGAGGAAGGGTACCTGT**AGAGAGCT**TCTTTTTGTTTCTACCTGGCCAAAGTTAGATGGGACAAAGATGAATGGCATGTCCCTTCTCTGAAGTCCGTTTGAGCAGAACTACCTGGTACCCCGAAAGAAAATCTTAGGCTACCACATTCTATTATTGAGAGCCTGAGATGTTAGCCATAGTGGACAAGGTTCCATTCACATGCTCATATGTTTATAAACTGTGTTTTGTAGAAGAAAAAGAATCATAACAATACAAACACACATTCATTCTCTCTTTTTCTCTCTACCATTCTCAACCTGTATTGGACAGCACTGCCTCTTTTGCTTACTTGCTGCCTGTTCAAACTGAGGTGGAATGCAGTGGTTCCCATGCTTAACAAATCATTAAAACACCCTAGAACACTCCTAGGATAGATTAATGTAGTAAGTCTGGCTAGGCCTTGTGTTGCCTTTTTTTTTTTTTTTTTTTTCTTTTCTTTTCTTCTCTTCCCATGCACTATTCTGGAGGTTTGCCAGGTTTGGGGAGACATGGAGTAAAAGAAAGATAGGCAACTCATGGATGGTGGGAGAGCAGGTAAGCAGGTCTGATCTCAAGGGCCCACATGAGGCATCAGTATATATTAGGCAGGTAGGGATCTCTGGCTTTGGTAATTCTTTATGAGAGGATCCTAGCCTTTGAAGCTGGGAGCAGGAGTCAGTGGCTACAGTGGGAAGGAGTACTGCAGGTTGGGGCCAAAGTGATACACAGCTTAGAAGGCAGCCTTCCTCCACTTACTCAACAAATCTTTATTTAGTGACTCTCCAAGTCCTAGTGATTATTATTATTGTTCACTCCACATTTGGCTTAATGGGTAATGCTATTACCCATTGCCTAACTAGGTTTGCAGTAGTGGAATCTCCAGAGATAGCAGGCTTAGTAAGCTGGAGGTAGGACATGAAGTCCCCCAAAACTTGATGTCCTATTTTTATGTGAGTTGGACAGTGGTTATCTTTTGCCTGTTGATATCTTAAAGCAGCAGAGTGGTATAGAAATTTGCGGTTATGACAGACCCGGGTTAAAAATCACAGCTGTGCCATTTGCTTTGATATTTTGAGCAAGGTAGCTAAATTTTCTGAGCTTCTATTTTCTCATCTGTAAAATGAGGATACGTACCTGTTCTTTTTTTTCTTTCTTTTTATTTCTTTTAGAGATAGGGTCTCGCTTTGTTGCCCAGGCTGGAGTGCACTGGCATGATCATGGCTCACTGCAGCCTCAAATTCCCAGGCTCAAGCAATCCTCCCACCTCAGCCTCCCCATTAGCTGGGACTACAGGGCCATGCCATCATGCCCAGCTAATTTAAACATAGTTTTCAGAGATGGAGCTCACTATGTTGCCCAGGCTGGTCTTGAATTCTTGGTCTCAAGCAATCCTCCCACTGCAGCCTTCCAAAGTGCTGGGCGTACAAGCGCAAGCCACTGTGCCCAGCTGTCAGACGCTGAGTTTTAATTATGCACCAAACTCCAGCCCGCAGATCCTCTTCACCAAAGCCCCTGGCTGGTCTAGCCCATCATGACTTCTCTAGGAACAGTCCTTCTTTAGGACTATAAAGTATTAACAAAAGTCTGTAGATTAAGGAGCCTGCATAAAGAATTCTGGATACAGGCCCCTGTCTTTCCAAAGTTCCTCTCCAATATCCCTTGGGGTCCTCATGTTTTTGAAGCAGCTTCACTCTGCACAGGCAGCAGGAGGTTGGGGGAGCCATAGCTCTGGGCCACGGGGGCAGATTTATTTGGATGATAGGACTAATATTTGTGTAACCTGCTG

Bold: the wildtype seed sequence for **miR-103a-2-5p**

Underline: the wildtype seed sequence for miR-660-3p

**Mutant 3’ UTR of WNT2B:**

ACACACAGATACCTCACTCATCCCTCCAATTCAAGCCTCTCAACTCAAAAGCACAAGATCCTTGCATGCACACCTTCCTCCACCCTCCACCCTGGGCTGCTACCGCTTCTATTTAAGGATGTAGAGAGTAATCCATAGGGACCATGGTGTCCTGGCTGGTTCCTTAGCCCTGGGAAGGAGTTGTCAGGGGATATAAGAAACTGAGCAAGCTCCCTGATTTCCCGCTCTGGAGATTTGAAGGGAGAGTAGAAGAGATAGGGGGTCTTTAGAGTGAAATGAGTTGCACTAAAGTACGTAGTTGAGGCTCCTTTTTTCTTTCCTTTGCACCAGCTTCCCGATACTTCTTGGTGTGCAAGAGGAAGGGTACCTGT**GCUGUAGC**TCTTTTTGTTTCTACCTGGCCAAAGTTAGATGGGACAAAGATGAATGGCATGTCCCTTCTCTGAAGTCCGTTTGAGCAGAACTACCTGGTACCCCGAAAGAAAATCTTAGGCTACCACATTCTATTATTGAGAGCCTGAGATGTTAGCCATAGTGGACAAGGTTCCATTCACATGCTCATATGTTTATAAACTGTGTTTTGTAGAAGAAAAAGAATCATAACAATACAAACACACATTCATTCTCTCTTTTTCTCTCTACCATTCTCAACCTGTATTGGACAGCACTGCCTCTTTTGCTTACTTGCTGCCTGTTCAAACTGAGGTGGAATGCAGTGGTTCCCATGCTTAACAAATCATTAAAACACCCTAGAACACTCCTAGGATAGATTAATGTAGTAAGTCTGGCTAGGCCTTGTGTTGCCTTTTTTTTTTTTTTTTTTTTCTTTTCTTTTCTTCTCTTCCCATGCACTATTCTGGAGGTTTGCCAGGTTTGGGGAGACATGGAGTAAAAGAAAGATAGGCAACTCATGGATGGTGGGAGAGCAGGTAAGCAGGTCTGATCTCAAGGGCCCACATGAGGCATCAGTATATATTAGGCAGGTAGGGATCTCTGGCTTTGGTAATTCTTTATGAGAGGATCCTAGCCTTTGAAGCTGGGAGCAGGAGTCAGTGGCTACAGTGGGAAGGAGTACTGCAGGTTGGGGCCAAAGTGATACACAGCTTAGAAGGCAGCCTTCCTCCACTTACTCAACAAATCTTTATTTAGTGACTCTCCAAGTCCTAGTGATTATTATTATTGTTCACTCCACATTTGGCTTAATGGGTAATGCTATTACCCATTGCCTAACTAGGTTTGCAGTAGTGGAATCTCCAGAGATAGCAGGCTTAGTAAGCTGGAGGTAGGACATGAAGTCCCCCAAAACTTGATGTCCTATTTTTATGTGAGTTGGACAGTGGTTATCTTTTGCCTGTTGATATCTTAAAGCAGCAGAGTGGTATAGAAATTTGCGGTTATGACAGACCCGGGTTAAAAATCACAGCTGTGCCATTTGCTTTGATATTTTGAGCAAGGTAGCTAAATTTTCTGAGCTTCTATTTTCTCATCTGTAAAATGAGGATACGTACCTGTTCTTTTTTTTCTTTCTTTTTATTTCTTTTAGAGATAGGGTCTCGCTTTGTTGCCCAGGCTGGAGTGCACTGGCATGATCATGGCTCACTGCAGCCTCAAATTCCCAGGCTCAAGCAATCCTCCCACCTCAGCCTCCCCATTAGCTGGGACTACAGGGCCATGCCATCATGCCCAGCTAATTTAAACATAGTTTTCAGAGATGGAGCTCACTATGTTGCCCAGGCTGGTCTTGAATTCTTGGTCTCAAGCAATCCTCCCACTGCAGCCTTCCAAAGTGCTGGGCGTACAAGCGCAAGCCACTGTGCCCAGCTGTCAGACGCTGAGTTTTAATTATGCACCAAACTCCAGCCCGCAGATCCTCTTCACCAAAGCCCCTGGCTGGTCTAGCCCATCATGACTTCTCTAGGAACAGTCCTTCTTTAGGACTATAAAGTATTAACAAAAGTCTGTAGATTAAGGAGCCTGCATAAAGAATTCTGGATACAGGCCCCTGTCTTTCCAAAGTTCCTCTCCAATATCCCTTGGGGTCCTCATGTTTTTGAAGCAGCTTCACTCTGCACAGGCAGGCATGCATTGGGGGAGCCATAGCTCTGGGCCACGGGGGCAGATTTATTTGGATGATAGGACTAATATTTGTGTAACCTGCTG

Bold: the mutant seed sequence for **miR-103a-2-5p**

Underline: the mutant seed sequence for miR-660-3p

**Table S4. The sequence of wildtype and mutant linear form of circRNA-SORE**

**Wildtype linear form of circRNA-SORE:**

CAGCAACAACTCCAGGCCCAGCATTTATCACATGGACATGGTCTCCCCGTACCTCTGACTCCACACCCTTCAGGGCTCCAGCCCCCTGCCATTCCACCCATCGGTAGCAGTGCCGGGCTTCTGGCCCTCTCCAGTGCTCTAGGAGGTCAGTCCCATCTTCCAATTAAAGATGAGA**AGAAGC**ACCATGACAATGATCACCAAAGAGACAGAGACTCCATCAAG

Bold: the wildtype target site of **miR-103a-2-5p**

Underline: the wildtype target site of miR-660-3p

**Mutation 1 of the linear form of circRNA-SORE:**

CAGCAACAACTCCAGGCCCAGCATTTATCACATGGACATGGTCTCCCCGTACCTCTGACTCCACACCCTTCAGGGCTCCAGCCCCCTGCCATTCCACCCATCGGTAGCAGTGCCGGGCTTCTGGCCCTCTCCAGTGCTCTAGGAGGTCAGTCCCATCTTCCAATTAAAGATGAGA**CTCTAT**ACCATGACAATGATCACCAAAGAGACAGAGACTCCATCAAG

Bold: the mutant target site of **miR-103a-2-5p**

Underline: the wildtype target site of miR-660-3p

**Mutation 2 of the linear form of circRNA-SORE:**

CAGCAACAACTCCAGGCCCAGCATTTATCACATGGACATGGTCTCCCCGTACCTCTGACTCCACACCCTTCAGGGCTCCAGCCCCCTGCCATTCCACCCATCGGTAGCAGTGCCGGGCTTCTGGCCCTCTCCAGTGCTCTTCACTATCAGTCCCATCTTCCAATTAAAGATGAGA**AGAAGC**ACCATGACAATGATCACCAAAGAGACAGAGACTCCATCAAG

Bold: the wildtype target site of **miR-103a-2-5p**

Underline: the mutant target site of miR-660-3p
